# Integrated multidisciplinary analysis of mobile digital radiographic acquisitions of the mummies of the Hermits from the Sanctuary of Madonna della Corona (Italy - 17th to 19th Century CE)

**DOI:** 10.3389/fmed.2024.1492328

**Published:** 2025-01-15

**Authors:** Omar Larentis, Ilaria Gorini, Michele Campus, Marta Lorenzetti, Giancarlo Mansueto, Susanna Bortolotto, Emanuele Zappa, Andrea Gregorini, Laura Rampazzi, Stefano Vanin, Giuseppina Carta, Alberto Carli, Lara Simonaitis, Lisa De Luca, Enrica Tonina

**Affiliations:** ^1^CROP - Centre of Research in Osteoarchaeology and Paleopathology, Biotechnologies and Life Sciences Department (DBSV), University of Insubria, Varese, Italy; ^2^LaBAAF - Bagolini Laboratory of Archaeology, Archaeometry, Photography, Department of Humanities, University of Trento, Trento, Italy; ^3^Superintendence for Archaeology, Fine Arts, and Landscape for the provinces of Como, Lecco, Monza-Brianza, Pavia, Sondrio and Varese, Milan, Italy; ^4^Fujifilm Italia, Cernusco sul Naviglio, Italy; ^5^Studio di conservazione tessile Lorenzetti, Verona, Italy; ^6^Diagnostics and Public Health Department (DDSP), University of Verona, Verona, Italy; ^7^Department of Architecture and Urban Studies, Polytechnic University of Milan, Milan, Italy; ^8^Department of Mechanical Engineering, Polytechnic University of Milan, Milan, Italy; ^9^Department of Human Sciences and Innovation for the Territory, University of Insubria, Como, Italy; ^10^Earth, Environment and Life Sciences Department (DISTAV), University of Genoa, Genoa, Italy; ^11^SUSeF, Department of Humanities, Social Sciences and Education, University of Molise, Campobasso, Italy

**Keywords:** X-ray analyses, anthropology, paleopathology, entomology, restoration, conservation, taphonomy, ethics

## Abstract

Paleoradiology has become a standard diagnostic method in the study of mummified or embalmed bodies. Among the various available techniques, computed tomography valuing for its ability to provide detailed information. However, computed tomography equipment is not always accessible to research teams, cannot be easily transported to all conservation sites, and raises health concerns. Therefore, mobile digital radiographic technology is often the most suitable investigative tool in specific contexts. In this study, three mummies preserved at the Sanctuary of Madonna della Corona, perched on a cliff above the Adige River Valley on the Monte Baldo at an elevation of 775 m a.s.l., were analyzed using radiographic techniques. The impossibility of transferring the bodies due to the sanctuary’s remote location imposed the use of lightweight, portable equipment for the radiological examination. This article highlights next-generation X-ray technology utility, potential, and limitations in investigating clothing fabrics, restoration history, taphonomy, entomology, anthropology, and paleopathology. The interdisciplinary approach in this study has unveiled new historical and biological insights about these mummies, which, though revered in popular devotion, had previously been unknown to both Italian and international scientific communities.

## 1 Introduction

The mummification of bodies of Saints or Blessed in Christianity, as well as other beliefs, is often the result of anthropogenic interventions aimed at their preservation, preparation, and display for promoting worship and devotion (1, 2). Over the centuries, these mummies have undergone relocation and restoration efforts to maintain their appearance or rectify anthropogenic or taphonomic damage (3, 4). External inspection of the bodies has significant limitations as it fails to analyze details beneath the skin surface, especially with clothed mummies (5). While some endoscopic techniques may provide internal insights, they are invasive, and there is a risk of compromising bodily preservation (6). Radiodiagnostics is a non-invasive, non-destructive investigative technique notable for its effectiveness, expediency, and capacity to address pivotal inquiries in mummies studies (7). Furthermore, employing radiation with modern apparatuses is safe for subsequent biomolecular analyses, such as ancient DNA examination (8).

Radiological analyses provide a wide array of biological information on subjects, including sex, age, pathologies, characteristics of human tissues, and data on the deposition context, such as taphonomic details (9). Additionally, they offer insights into the “life history” of mummified subjects: they encompass information on actual preservation, strategies employed for a future conservation project, the presence of foreign bodies, attacks by infesting mold, insects, or larger animals that have sought refuge and habitat within the bodies over time (e.g., mice or rats). Advanced technologies, like computed tomography (CT), further allow detailed three-dimensional representations useful for outreach purposes, such as facial approximation (10, 11).

However, research on mummies must address several issues regarding CT techniques. Hospital machinery may be time- and resource-intensive, perceived as conflicting with clinical needs. Mummies must be transported to the CT facility as these apparatuses are often non-portable. Even with access to a portable unit, its size may render it unsuitable for transportation into hard-to-reach locations. Handling of the subjects can also determine problems of preservation and safety: every movement may damage the remains, and the bodies may serve as vectors for the spread of harmful biological agents to health (12).

**Figure 1:**
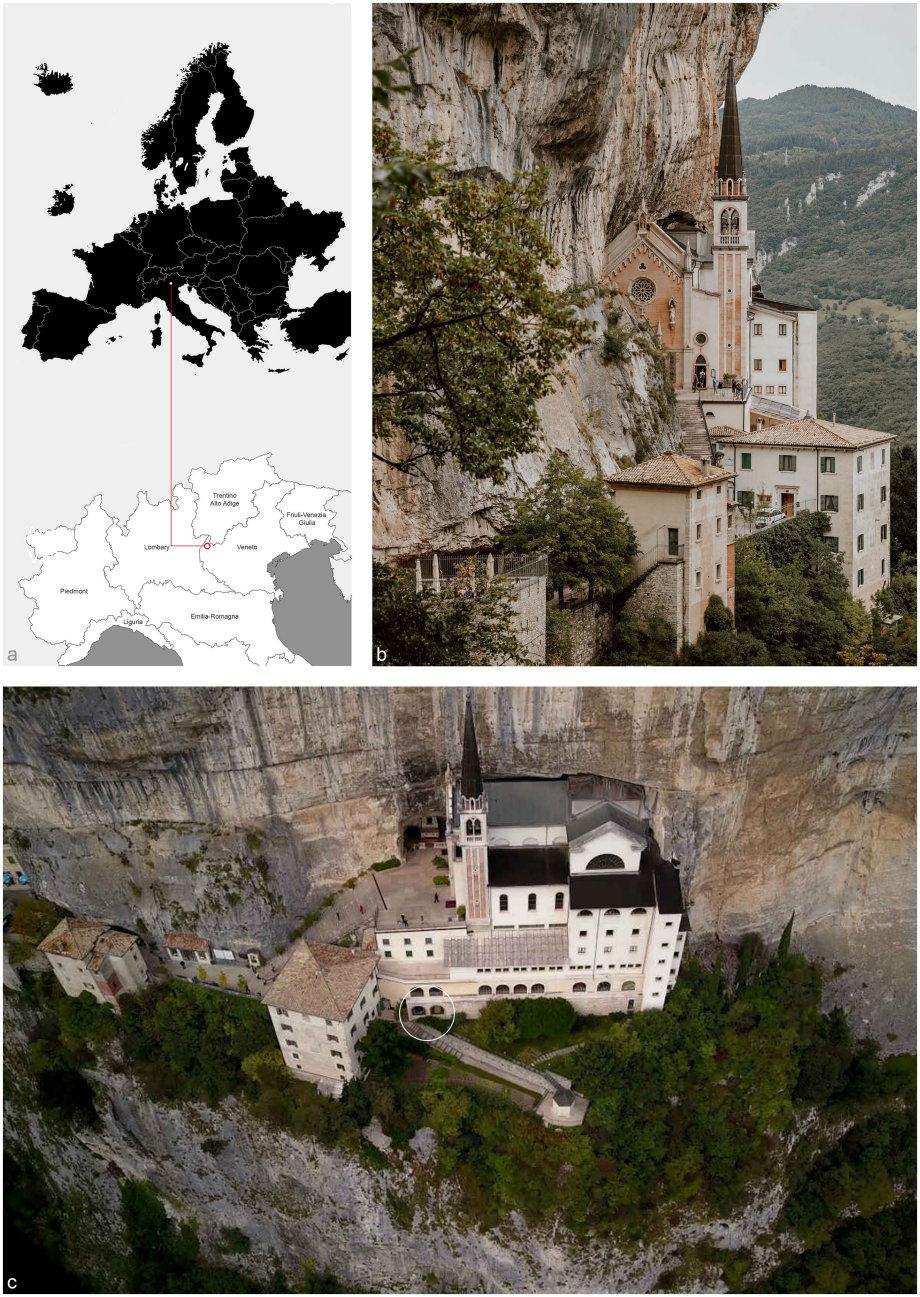
(a) The Sanctuary of Madonna della Corona is in Northern Italy, in Ferrara del Baldo in the western Veneto Prealps, latitude: 45.648155|longitude: 10.854965; (b) The sanctuary is built on a sheer cliff face of Mount Baldo, overlooking the valley of the Adige River; (c) The white circle marks the hermits’ burial area, where researchers set up the field laboratory.

Sudden fluctuations in humidity and temperature can also lead to abrupt changes in relative humidity, a crucial parameter for the stability of remains, resulting in dehydration, causing mechanical stress, or rehydration, leading to potential biochemical and biological damages (13). These variables need a thorough evaluation by research teams, which requires adequate funding to cover secure handling, insurance, conservator involvement during transit, and any necessary preliminary sanitization operations. These substantial costs limit such practices. Furthermore, it should be noted that holy bodies hold significant symbolic importance for religious communities: their movement may raise negative feelings among the worshipping community (1–4). Consequently, acquiring radiographs on-site may be preferable when transporting mummies or installing CT equipment is impractical or undesirable. In these contexts, it is important to assess the limitations and potentials of radiographic acquisitions considering anthropological, paleopathological, conservation, taphonomic, entomological, and ethical disciplines. This paper presents the investigation of three mummified bodies from the Sanctuary of Madonna della Corona (Figure 1), where researchers conducted X-ray imaging with a portable instrument as an initial step before further scientific analyses.

## 2 Materials and methods

**Figure 2:**
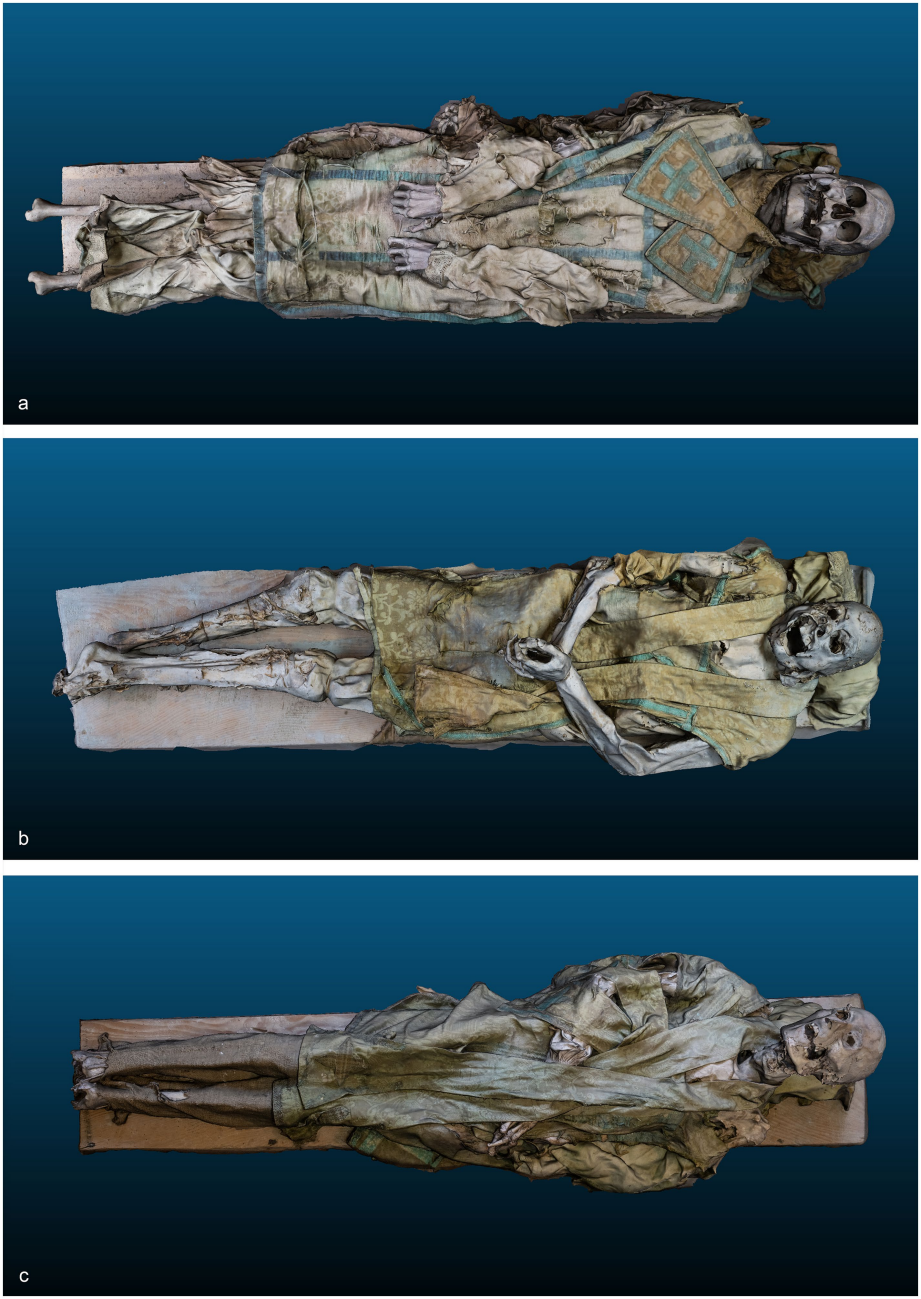
(a) Three-dimensional model of ID1; (b) Three-dimensional model of ID2; (c) Three-dimensional model of ID3.

This study focuses on three well-preserved mummified individuals buried in the Sanctuary of Madonna della Corona in Ferrara del Baldo (Verona, Northern Italy), identified as Individual 1 (ID1), Individual 2 (ID2), and Individual 3 (ID3) (Figure 2).

### 2.1 Context of conservation of the materials

Despite the first written evidence of hermits in the area dating to 1193 (14), the Madonna della Corona Sanctuary has likely more ancient origins, dating back to the phenomenon of hermitic-monastic settlement in the early Middle Ages, well-known in the Alps. Initially dedicated to Santa Maria di Monte Baldo, the church is documented in the 13th century but may have existed as early as the 12th century as a protective structure over a cave used as a place of prayer by the early monks practicing hermitage. During the late Middle Ages, a new church was built above the cave, while an underground chamber became a sepulcher for the hermits. The presence of these hermits is documented until 1598 thanks to a funerary slab commemorating the burial of the last of them preserved in the Sanctuary. Over the centuries, people expanded the sepulcher to accommodate the burial of both laypeople and religious figures. In 1932, an examination of the sepulcher provided the first written confirmation of the mummies. Subsequently, in 1975–1978, the Sanctuary was demolished and rebuilt. The demolition of the tomb oratory and the hermits’ sepulcher made way for new structures, including the area that now hosts the exhibition of the three bodies. Worshippers can see the bodies in wooden coffins with glass panels. These wooden structures have served their purpose since the 1930s, but their craftsmanship likely dates back even further, possibly to the late 19th century. The coffins do not provide an airtight seal, so the conservation parameters reflect those of the environment.

### 2.2 Methods

Digital photogrammetry allows the 3D reconstruction of the three mummies. This technique permits the 3D reconstruction of the surface of the target, relying on a sequence of 2D digital photography acquired from different and unknown points of view. The reconstruction of the 3D model used feature matches between different images of the target. To achieve a necessary field overlap. Each of the three individuals required multiple images to ensure good field overlap. 323, 403, and 413 images were captured and processed for ID1, ID2, and ID3, respectively. The digital camera used in this work is a Fujifilm X-T30, with a sensor resolution of 6246 × 4170 pixels and the following settings: ISO-2000, f/4, and a focal length of 18 mm. The used photogrammetry software is Agisoft Metashape Professional. The obtained 3D models are made of 157175 points for ID1, 403681 points for ID2, and 234827 points for ID3. A Fuji FDR Xair mobile digital radiographic system was placed directly in the storage area with the three bodies.

These enclosures have made it impossible to remove the bodies without risking potential damage, hindering both macroscopic inspection and radiological analysis. The detector, the radiological device that captures the X-ray beam from the FDR Xair tube and transmits the images to the console, was positioned directly under or laterally to the bodies for anteroposterior or lateral views; in detail, the FDR Xair’s detector, the FDR D-Evo III, is based on a CsI scintillator and specific licenses from Fujifilm, like a particular position of the TFT layer, the film-based technology that implements plastic support instead of glass support improving the sensibility of the detector to the low doses, and dedicated image post-processing; all of these peculiarities involve in high spatial and contrast resolution. The following routine projections and examination parameters were employed: cranium/cervical 62 kV – 1.6 mAs; thoracic/dorsal spine 64 kV – 2 mAs; pelvis/lumbar spine 64 kV – 2 mAs; lower limbs 60 kV – 1.6 mAs; upper limbs/shoulder 58 kV – 1.6 mAs.

After the X-ray acquisition, bodies underwent an anthropological examination to ascertain their age, sex, and bioanthropological characteristics (15). This examination involved observing skull morphology (16), pelvis morphology and their measurements (17), and inspecting external genitalia. Age-at-death estimation primarily relied on dental wear (18) and assessment of cranial suture closure (19), as soft tissues covered traditional diagnostic areas such as the pubic symphysis (20). For this reason, the estimates are categorized into broad age ranges rather than short age intervals, thereby increasing the potential for accuracy. An entomological survey was carried out, along with the state of degradation or decay and the possible conservation interventions (21). Finally, the team also valued the historical liturgical vestments of the subjects. Following radiological analysis, it was possible to integrate anthropological data with skeletal maturation assessment for age estimation and morphometric evaluation of the pelvis for sex determination, as with pathological data for each subject, discussed in paleopathological (22) and clinical literature.

Anthropologists conducted the investigations following the guidelines of the Italian Central Institute for Archaeology (*Istituto Centrale per l’Archeologia, ICA*) and the Italian Central Institute for Catalogues and Documentation (*Istituto Centrale per il Catologo e la Documentazione, ICCD*) (23) and were formally authorized by the Ministry of Culture (*Ministero della Cultura*, *MiC*).

## 3 Results

The results are organized into sections based on the relevant fields of expertise, further emphasizing the contributions that each discipline has gained using conventional radiography. The description begins with the historical aspects of the deceased, followed by details concerning their conservation and preservation, and finally, it addresses the biological characteristics that distinguish them.

### 3.1 Textile analyses

**Figure 3:**
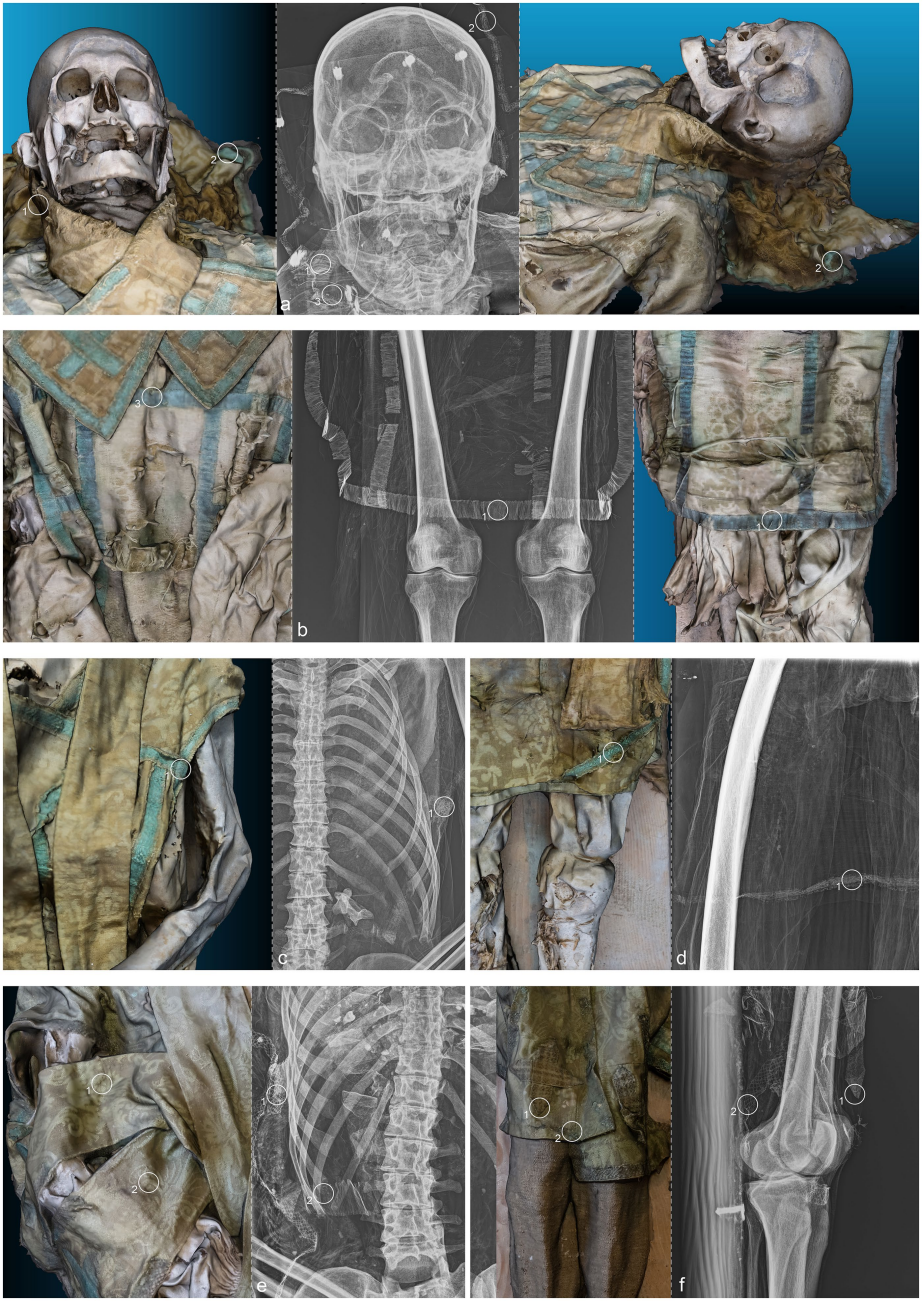
(a) Anteroposterior radiograph of the skull of ID1; (b) Anteroposterior radiograph of the lower limbs of ID1; (c) Anteroposterior radiograph of the thorax of ID2; (d) Anteroposterior radiograph of the right femur of ID2; (e) Anteroposterior radiograph of the thorax of ID3; (f) Lateral radiograph of the lower limbs of ID3. The three-dimensional reconstructions, separated by a dashed line from the radiographic acquisitions, correspond to the radiographed anatomical areas and are numbered accordingly.

ID1. The liturgical vestments of ID1 consist of a chasuble, a stole, an alb, and a pillow cover. Artisans crafted the chasuble’s passementerie with two distinct threads: an oxidized metallic thread and a silk thread (Figure 3a.1,2). X-ray analysis revealed the intricate weaving pattern running along the entire perimeter of the chasuble (Figure 3a.2). The distinctive feature identified was the use of metallic foil as the weft; the warp, presumably organic, was not detected (Figure 3a.1). The density of the weave and the loss of metallic foil due to significant oxidation were observed, with small fragments identified as fibrils. Fabric folds caused irregularities in the X-ray images. The passementerie on the maniple was likely woven on a loom, with a metallic thread warp (possibly with a silk core) and an undetermined weft (Figure 3a.3). The passementerie on the pillow cover exhibited a different weaving technique compared to the chasuble. A metallic foil, twisted around what appears to be a silk thread core, runs through both the warp and weft. Irregularities in the X-ray images were again due to fabric folds (Figure 3b.1).

ID2. The liturgical vestments of ID2 consist of a chasuble, a stole, a pillow cover, and what might be a maniple. The passementerie on the chasuble and stole was made with bobbin lace using two threads: an oxidized metallic foil and a silk thread. X-ray analysis revealed the metallic foil twisted around an organic thread core, present in both the warp and the weft (Figure 3c.1). The weave was more complex than that of the first exhibit, with a decorative pattern of alternating rhombuses and small lozenges. The radiographic analysis highlighted the intricate technique, which is difficult to discern with the naked eye due to significant oxidation of the metallic thread (Figure 3d.1).

ID3. The liturgical vestments of ID3 consist of a chasuble, a stole, a maniple, an alb, and footwear. A loom set to create a herringbone pattern wove two threads into the passementerie adorning the chasuble stole and maniple (Figure 3e.1,2): a partially oxidized gold metallic foil and a silk thread. Additionally, there was bobbin lace with a gold foil twisted around a silk core, featuring a net motif with undulating terminations (partially oxidized). X-ray analysis detected two different weaving techniques. The first, visible only in the weft direction, is a metallic foil twisted around an organic thread. The galloon, primarily made of organic material, did not reveal the weaving pattern through radiographic analysis. The second type of passementerie, clearly visible, showed a net motif entirely composed of twisted metallic foil (Figure 3f.1,2).

### 3.2 Taphonomic analyses

ID1. In the neurocranium, fine material occupies the base, partially filling the occipital region in a layer parallel to the deposition plane (Figure 4.1,5 green). The left frontozygomatic suture is notably more pronounced than the contralateral side due to a slight clockwise rotation of the frontal bone away from the zygomatic bone (Figure 4.2 green). The mandible is also displaced from its anatomical position, with a counterclockwise rotation, pivoting on the mandibular condyle and resulting in the dislocation of the contralateral side (Figure 4.3,6 green). All teeth are absent except for the left upper second premolar (Figure 4.7 green). Other teeth are visible on the floor of the nasopharynx and within the thoracic cavity, to the left of the tenth and eleventh thoracic vertebrae (Figure 4.4,8–10 green). The proximal phalanx of the right thumb is not in anatomical position (Figure 4.11 green), and both distal epiphyses of the fibulae are lacking (Figure 4.12,13 green).

**Figure 4:**
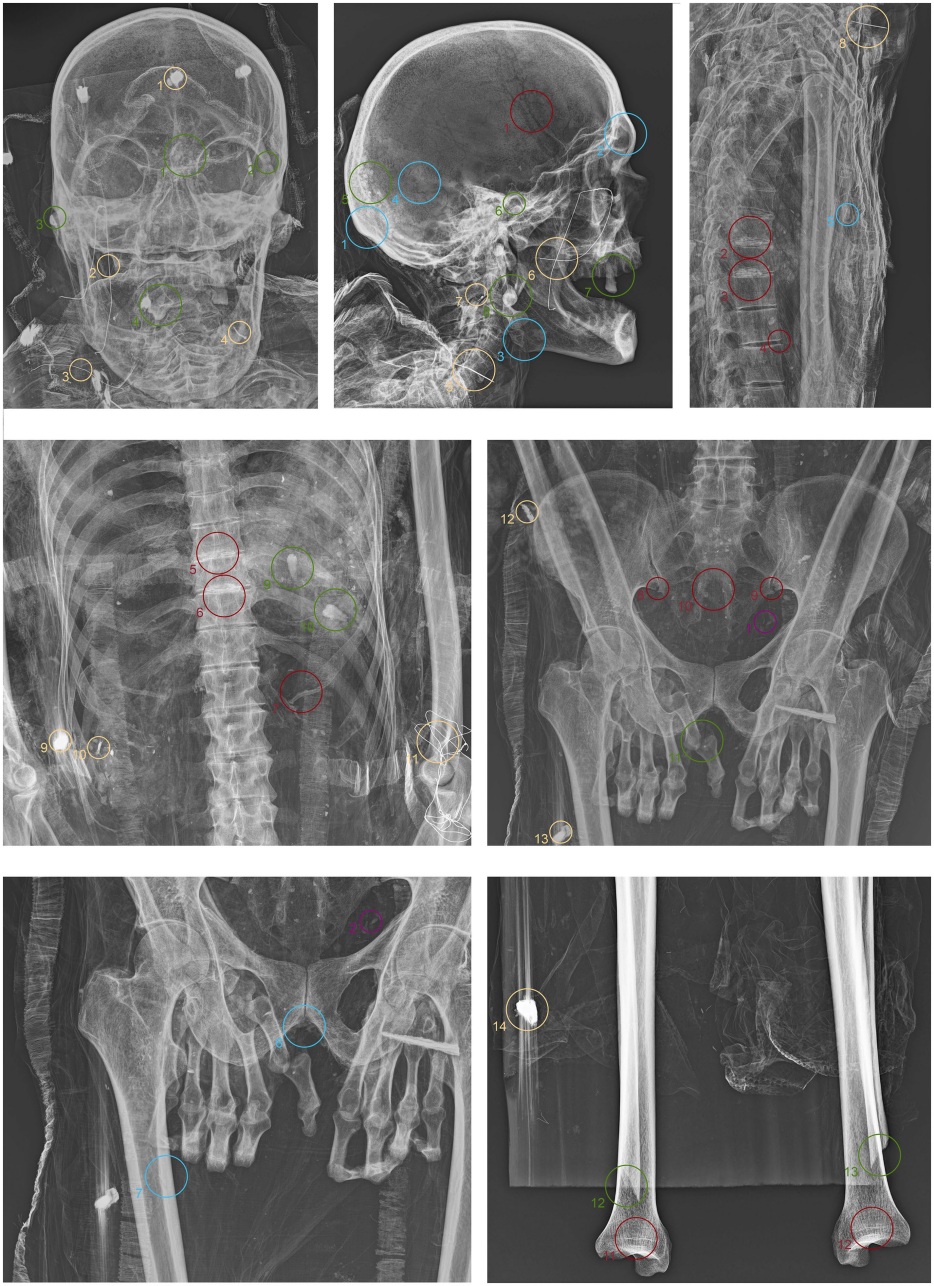
Anteroposterior and lateral radiographic acquisitions of the individual ID1. Green highlights indicate areas of interest for taphonomic study, yellow highlights indicate areas relevant to the assessment of restorations predating this study, violet is used for entomological analysis, blue for anthropological aspects, and red for paleopathological evidence.

ID2. In this subject, fine material is detectable in the occipital region, forming a single layer parallel to the mummy’s deposition axis (Figure 5.1 green). The cervical vertebrae and the first thoracic vertebra are no longer in anatomical connection, disrupting the continuity of the spinal column (Figure 5.2 green). They have migrated beneath the right side of the thorax at the mid-thoracic level, extending down to the lumbar region (Figure 5.5,6,10 green). Additionally, some teeth are not in an anatomical position—one within the thoracic cavity and another in the pelvic area (Figure 5.3,4,7,11 green). The sternum has migrated to the lower right thoracic area, and a medial phalanx is also displaced (Figure 5.8,12 green). The left clavicle is more vertical compared to the contralateral side, and, in addition, there is a slight retraction of the arm and forearm (Figure 5.9 green). The sacroiliac articular surface is particularly prominent, as is the pubic symphysis, exposed due to the separation of the two halves (Figure 5.13; Figure 6.1 green). The right femur is also rotated laterally by about 90° (Figure 5.14 green), causing lateral rotation of the knee and tibia. Notably, a fold in the soft tissues of the left thigh is evident at the level of the distal diaphysis of the left femur (Figure 6.2–4 green).

**Figure 5:**
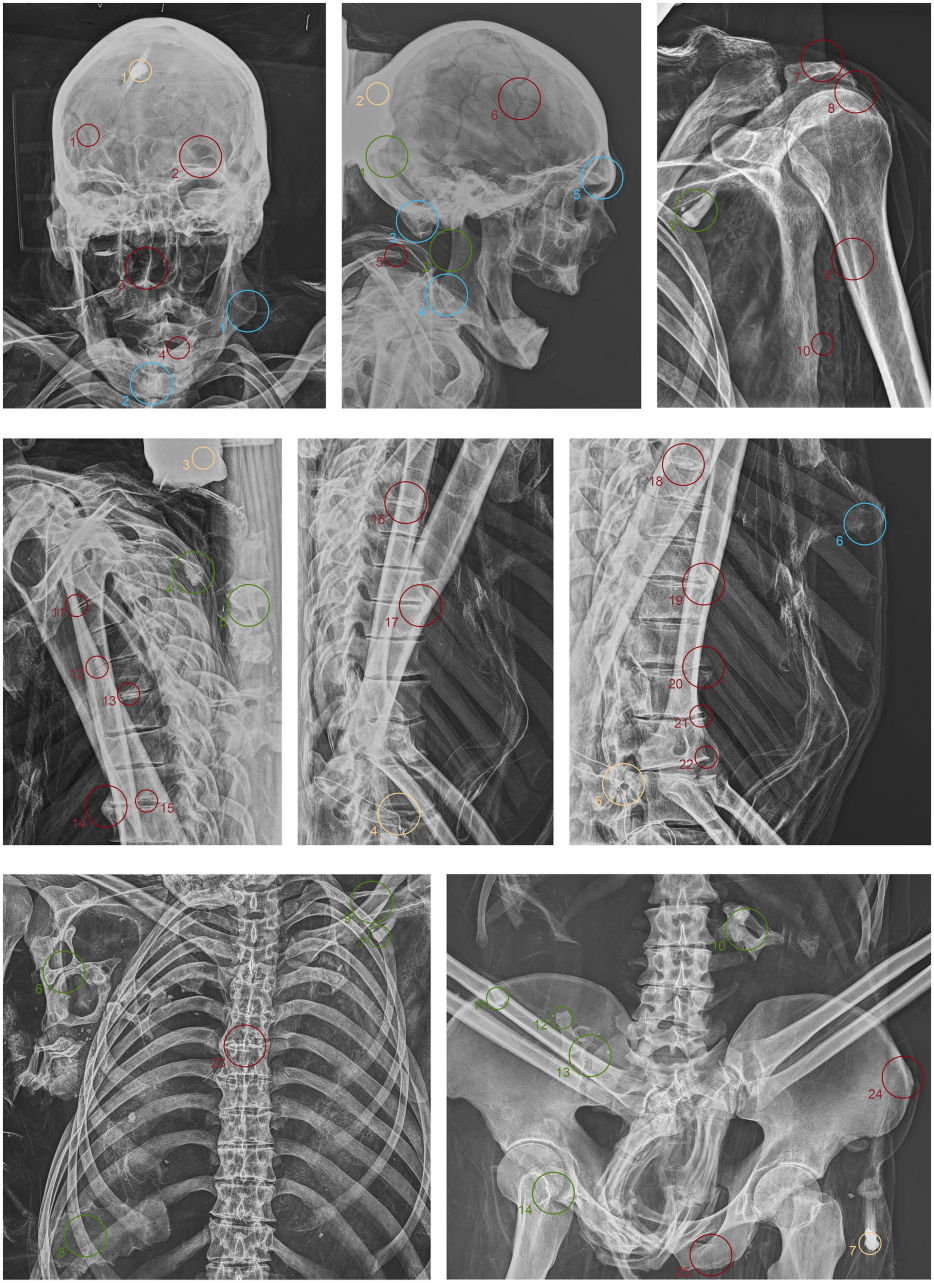
Anteroposterior and lateral radiographic acquisitions of the pelvis and upper limb of the individual ID2. Green highlights indicate areas of interest for taphonomic study, yellow highlights indicate areas relevant to the assessment of restorations predating this study, violet is used for entomological analysis, blue for anthropological aspects, and red for paleopathological evidence.

**Figure 6:**
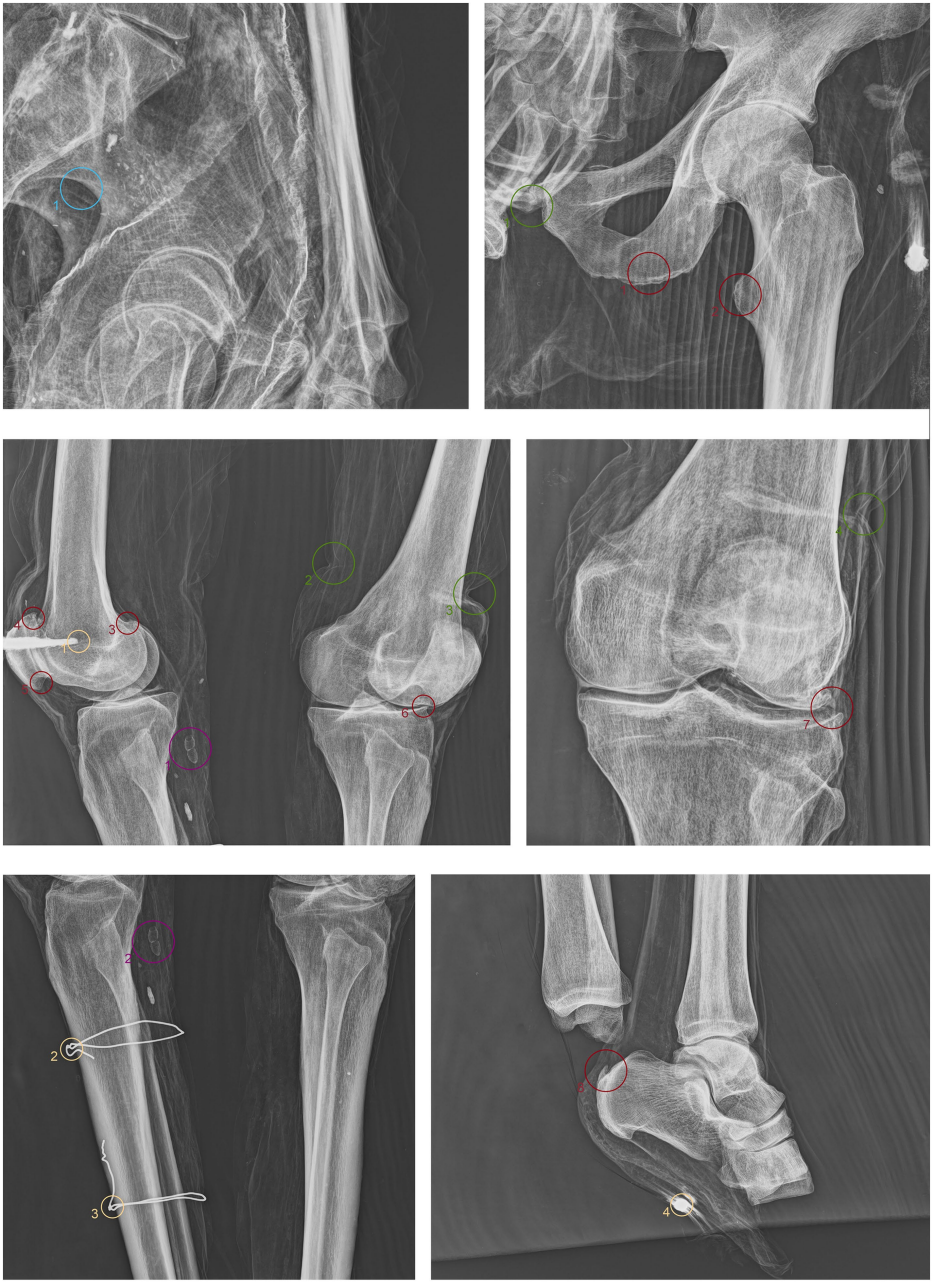
Anteroposterior and lateral radiographic acquisitions of the pelvis and lower limb of individual ID2. Green highlights indicate areas of interest for taphonomic study, yellow highlights indicate areas relevant to the assessment of restorations predating this study, violet is used for entomological analysis, blue for anthropological aspects, and red for paleopathological evidence.

ID3. As in the previous subjects, fine material is observed within the occipital region of the neurocranium (Figure 7.1 green). The hyoid bone is visible in its anatomical position (Figure 7.2 green). The upper spine remains in perfect anatomical alignment. However, there is a discontinuity between the first and second thoracic vertebrae, with the upper segment displaced anteriorly by approximately 4 cm in the sagittal plane (Figure 7.3,4 green). A bone fragment and the distal portion of the right fibula are recognized as fragmented at the level of the mid-diaphysis of the right femur (Figure 7.5,6 green).

**Figure 7:**
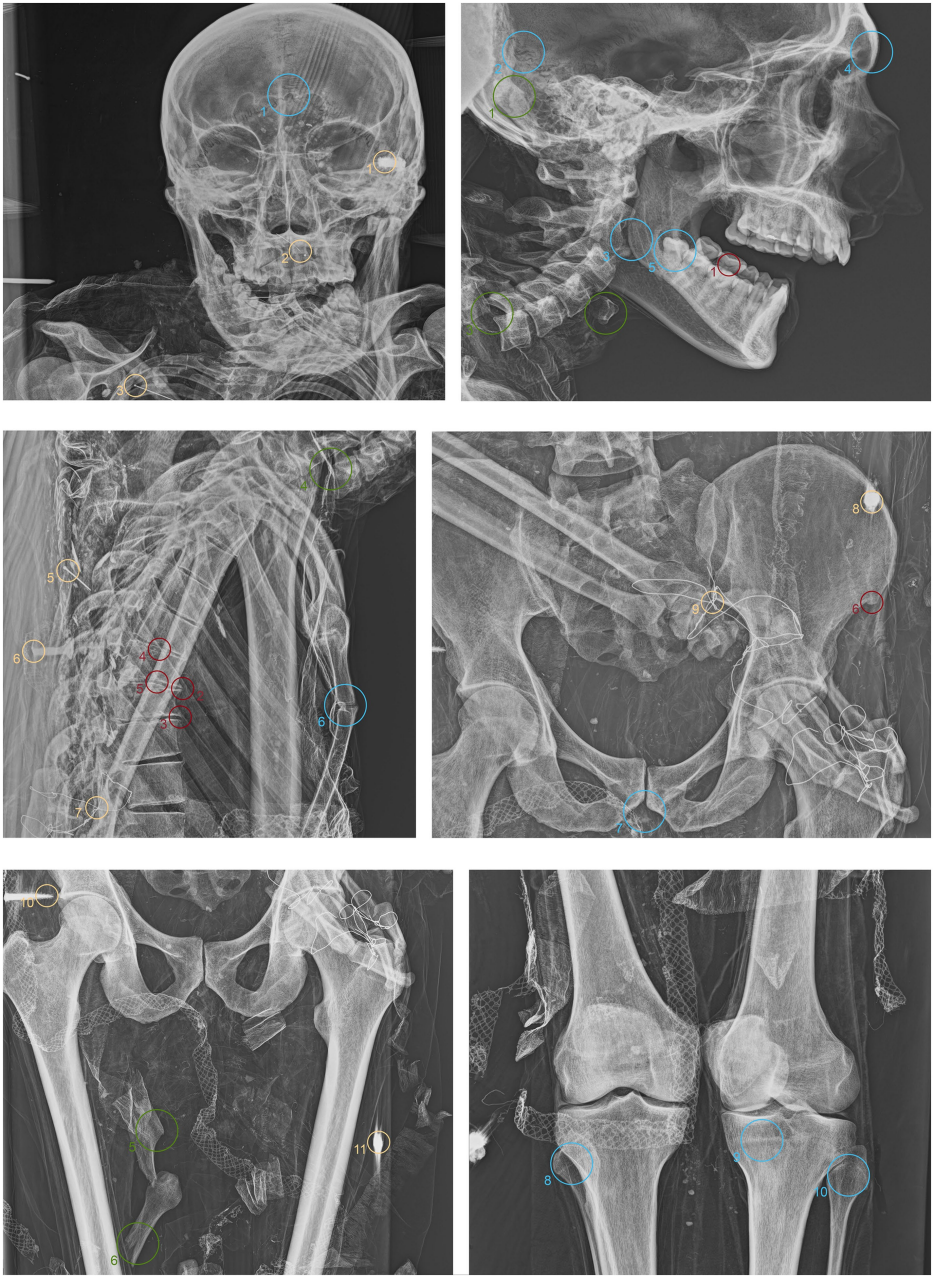
Anteroposterior and lateral radiographic acquisitions of the individual ID3. Green highlights indicate areas of interest for taphonomic study, yellow highlights indicate areas relevant to the assessment of restorations predating this study, violet is used for entomological analysis, blue for anthropological aspects, and red for paleopathological evidence.

### 3.3 Artifact analyses

ID1. The metal nails within the wooden support boards for the mummy are visible (Figure 4.1,9,13,14 yellow). Metal wire appears on various sections of the body; on the mandible, the wire appears unfastened, and numerous fragments of similar wire are visible at the shoulder level (Figure 4.2–8 yellow). Several loops of metal wire connect the left humerus, ulna, and radius, loosely knotted at the elbow (Figure 4.10–12 yellow).

ID2. Similarly, the metal nails used to assemble the wooden bases housing the bodies are visible (Figure 5.1,7; Figure 6.1,4 yellow). A particularly radiodense rectangular element wraps around the occipital bone beneath the skull (Figure 5.2,3 yellow). As in the previously discussed subject, metal wire was used to wrap around the left elbow (Figure 5.4,5 yellow) and to bind and tighten the mummified tissues of the right leg (Figure 6.2,3 yellow).

ID3. In this individual, numerous nail used for the mummy supports are visible (Figure 7.1,6,8,11 yellow). In this case, one image highlights the more radiodense halo that characterizes many of the metallic elements of the supports (Figure 7.10 yellow). Several small pins with heads are positioned in the upper part of the subject (Figure 7.2,3,5 yellow), while iron wire binds the right wrist and hand, with loops extending to bind individual fingers (Figure 7.7,9 yellow).

### 3.4 Entomological analyses

The search for entomological evidence from the X-rays carried out on the three bodies revealed only a few interesting elements, in contrast to the data obtained from the hand collection of insects and their fragments from the bodies (data not published). In particular, a pair of barrel-like elements of a size (<1 cm) compatible with puparia are visible on ID1 at the pelvis cavity (Figure 4.1,2 violet). The elements are radio-dense and can be interpreted as whole puparia, with the dead immature insects inside or with rat/mouse excrements. In addition, two amphora-like elements are detectable on the leg of ID2 (Figure 6.1,2 violet). These elements are comparable with black mud-dauber wasps’ nests, however, due to their position no direct observations were carried out. So, this remains a potential hypothesis.

### 3.5 Anthropological analyses

ID1. In the lateral view, the pronounced glabella, well-defined nuchal crest, and the right angle formed by the mandibular ramus and body are evident (Figure 4.1–3 blue). The lambda suture appears completely open (Figure 4.4 blue). The sternocostal surfaces of the ribs are well-defined and free from deformities, erosions, or marginal sclerosis (Figure 4.5 blue). The sciatic notch is narrow, and the subpubic angle is less than 90° (Figure 4.6 blue). The cortical thickness of both femoral diaphyses suggests robust bone trophism (Figure 4.7 blue).

ID2. In the anteroposterior view, a big gonial eversion and a robust mental symphysis are evident, with a prominent mental protuberance and pronounced mental tubercles (Figure 5.1,2 blue). In the lateral view, the pronounced glabella, robust mastoid processes, and relatively open mandibular angle are visible (Figure 5.3–5 blue). The sternocostal surfaces of the ribs show deformities, erosions, and marginal sclerosis (Figure 5.6 blue). Both the lateral and anteroposterior views reveal a narrow sciatic notch (Figure 6.1 blue).

ID3. In both anteroposterior and lateral views, the sagittal suture and lambda are unfused (Figure 7.1,2 blue). The subject has a prominent gonion of the mandible, a mandibular angle near 90°, and a very pronounced glabella (Figure 7.3,4 blue). The presence of the right lower third molar is also evident, while the other three third molars are absent (Figure 7.5 blue). While the bones have fused, the manubrium and first sternebra remain separate, and fusion lines on the proximal ends of the fibulae and tibiae are still evident (Figure 7.6, 8–10 blue). The pelvis is robust, with a subpubic angle of approximately 90°, although the left hemipelvis is slightly rotated counterclockwise (Figure 7.7 blue).

### 3.6 Paleopathological analyses

ID1. X-ray lateral projection analysis of the skull reveals areas of reduced radiodensity that follow the course of the middle meningeal artery, suggesting the presence of a deep groove corresponding to the vessel’s anatomy (Figure 4.1 red). ID1. The X-ray lateral projection of the skull shows areas of reduced radiodensity along the path of the middle meningeal artery, indicating a pronounced groove that aligns with the artery’s course (Figure 4.1 red). In the lower thoracic spine, there are calcifications of the intervertebral space between the 8th and 11th vertebrae, more expressed toward the lumbar region and particularly evident in the lateral projection (Figure 4.2,3,5,6 red). This condition is not associated with marginal lipping on the edges of the vertebral bodies, nor are there any signs of osteolytic activity on the superior or inferior surfaces of the vertebrae (Figure 4.4 red). Calcifications are also visible starting from the articular sternal surface of ribs (Figure 4.7 red). Heterotopic ossification is observed in the pelvic area, corresponding to the sacroiliac joint surface. The anteroposterior projection of this anatomical region reveals osteophytes along the inferior margins of the joint in the ventral view (Figure 4.8,9 red). The non-closure of the sacral laminae up to S3 is also documented (Figure 4.19 red). The densification of bone tissue is observed in the distal diaphysis of both tibiae, approximately 1.7 cm from the distal articular surface (Figure 4.11,12 red).

ID2. The skull shows a dense network of venous impressions on the parietal bones, particularly visible in the anteroposterior projection (Figure 5.1,6 red), along with the presence of several Pacchioni’s foramina. The frontal sinuses expand laterally, almost reaching above the orbits (Figure 5.2 red). The anterior nasal spine appears slightly rotated to the right relative to its anatomical axis (Figure 5.3 red). This individual exhibit complete edentulism, associated with mature alveolar bone retracted from the alveolar region (Figure 5.4 red). Proceeding to the analysis of the appendicular skeleton, the acromion of the left clavicle shows marked roughness at the insertion area of the trapezius muscle, as at the origin of the subscapularis muscle (Figure 5.7 red). The joints between the humerus and scapula exhibit greater radiodensity (Figure 5.8 red). An area free of bone overlap allows the observation of increased radiodensity at the insertion of the pectoralis major muscle and inside the humerus-scapular joint (Figure 5.9 red). Heterotopic ossification is also noted on the margin of the lateral border of the scapula (Figure 5.10 red). The thoracic spine presents intervertebral calcification between the 5th and 6th vertebrae (Figure 5.13,16,18,23 red), fusion of the 8th and 9th vertebrae through a dense bony bridge (Figure 5.14,17,19 red), and intense osteophytic change along the margin of the entire segment, particularly evident between the 9th and 11th thoracic vertebrae (Figure 5.11,12,20–22 red). Sclerosis is observed in the superior and inferior vertebral articular surfaces, accompanied by granular and concave areas in the vertebral bodies (Figure 5.15 red). Osteophytes are observed at the insertion points of the oblique muscles on the iliac crest, at the various muscles originating from the ischial tuberosity, and at the insertion area of the iliopsoas muscle during the analysis of the pelvic girdle (Figure 5.24,25; Figure 6.1,2 red). The origin of the gastrocnemius muscle is also particularly pronounced (Figure 6.3 red). At the level of the knee joint, we noticed osteophytes on the margin of the right patella (Figure 6.4,5 red), both on the superior and inferior portions of the joint, as well as on the lateral articular margin of the distal femur and proximal tibia, with productive changes that have significantly altered the morphology of the joint (Figure 6.6,7 red). It is worth mentioning that osteophytes are also present at the insertion of the Achilles’ tendon (Figure 6.8 red).

ID3. The lateral projection of the skull highlights the morphology of the pulp chamber of the left second lower molar, which exhibits a “chair-shaped” configuration (Figure 7.1 red). The 8th thoracic vertebra is compressed in the anterior view, accompanied by a milder compression of the 7th vertebra. This condition is associated with osteophytes along the margins of both vertebral bodies, as well as the preceding and following vertebrae (Figure 7.2,3 red). Additionally, the vertebral endplates, which are slightly sclerotic, show small areas of erosion in the central portion (Figure 7.4,5 red). In this individual, the insertions of the oblique muscles on the iliac crest also show significant alterations in this region (Figure 7.6 red).

## 4 Discussion

The radiographic investigations of liturgical vestments have revealed the diverse treatments of metallic foils and the density of the textile weave. The possibility of identifying metallic foils, silk threads, and the effect of oxidation highlights the importance of advanced imaging techniques in preserving and studying historical garments (24, 25). The variations in weaving techniques and material compositions among the clothes reflect the diverse methods of textile production used during the examined period (26, 27). The research team was able to establish a chronological order for the bodies thanks to these differences: ID1 dates to the early 19th century, ID2 to the mid-17th century, and ID3 to the 18th century. The X-ray images highlighted the intricate weaving patterns, showing the use of metallic foil twisted around an organic thread core, present in both the warp and the weft. In some cases, the metallic threads were heavily oxidized, leading to the formation of fibrils and contributing to the irregularities observed in the X-ray images, which were often due to fabric folds. This observation leads us to hypothesize a local or Venetian production for all three cases.

Previous studies have shown that X-ray imaging can effectively reveal hidden details in ancient textiles, such as the weave density, thread composition, and the extent of degradation due to environmental factors (28–30). In the case of the liturgical vestments analyzed in this study, the advanced imaging techniques confirmed the presence of complex weaving patterns and highlighted the significant role of metallic foils in the overall design. Moreover, the use of X-ray diagnostics to assess the conservation conditions of these historical textiles offers a non-invasive method to monitor and preserve these valuable artifacts. Visualizing areas not accessible to the naked eye allows for a more comprehensive understanding of the state of preservation and informs targeted restoration efforts (31).

Concerning the remains’ analyses, radiographic imaging revealed significant bone dislocations, including the displacement of the mandible in ID1 and ID3 and the proximal phalanx of the right thumb in ID1. In the case of ID1, the mandibular bone was repositioned and secured with metal wire to establish a lasting anatomical connection. The left elbow of the same subject, of ID2, as the hand bones of ID3, show the same treatment. In all cases, these connections are vulnerable to dislocation from their anatomical positions once the mummified soft tissues deteriorate, which indicates that these dislocations are not related to events occurring during the mummification process (32–34). Another commonality among these repairs is that they were performed in easily accessible body areas and were relatively simple to address. These interventions appear rudimentary, often showing a lack of durability, likely the result of routine maintenance practices aimed at preserving the bodies for devotional purposes (35).

Additional dislocations highlight how this imaging technique can document the extent of skeletal displacements and assist in reconstructing the sequence of taphonomic events (36).

The loss of numerous dental elements, often found within the body cavities due to gravity, multiple dislocated vertebrae, and the sternum in one case, suggest episodes of abrupt movement affecting the mummies, compromising their structural integrity. This is particularly evident in ID2, where the absence of cervical vertebrae results in significant head mobility. Similarly, ID3 exhibits a clear discontinuity in the upper spine, which would not have been visible to the naked eye due to the overlying mummified soft tissues that obscure that area. The same observation applies to the pelvic regions of ID2 and ID3, which display a significant loss of perfect anatomical articulation, hidden from view by the soft tissues.

The medio-lateral rotation of the right leg in ID2, on the other hand, can be attributed to a movement that occurred during the mummification process. This individual’s legs were tightly bound in multiple places, a technique likely related to his heavyweight to immobilize the body or facilitate its transport. The restoration intervention on this leg was probably intended to maintain the cohesion of the bulky soft tissue mass of the limb following its partial deterioration.

Other cases, such as the fractures of the distal diaphyses of the fibulae in ID3, illustrate the violent nature of certain post-mortem manipulations, which likely resulted in the concealment of these bone fractures beneath the subject’s liturgical vestments. Other cases, such as the fractures of the distal diaphyses of the fibulae in ID3, illustrate the violent nature of post-mortem manipulations, which likely resulted in the concealment of these bone fractures beneath the subject’s liturgical vestments. From a taphonomic perspective, fine material within the neurocranium of the subjects is consistently located in the occipital region, aligned parallel to the bodies’ axis. This material likely represents the remnants of the neurocranial soft tissues, suggesting that mummification occurred while the body was horizontal (37, 38).

The radiographic analysis also revealed the presence of metal nails within the boards under the bodies. This finding helps in understanding the construction process of the supports. The varying shapes and sizes of the nails, along with their irregular positioning, suggest a non-professional assembly of the supports, driven more by necessity than by a desire to create an aesthetically pleasing or high-quality base (39).

The paucity of entomological evidence detected by the X-rays can be explained by a technical limitation of the method: on mummified bodies, the most abundant insect traces are represented by empty puparia, in which the thin cuticle is X-ray-transparent (40, 41). In addition, other elements such as beetle fragments, mainly belonging to mold-feeders (*Crystophagus, Dinerella, Latridium*, etc.) are too small to be detected. Difficult to visualize, Lepidoptera cocoons are often associated with mummified bodies (42).

The anthropological inspection allowed for the evaluation of the genital area of ID1 and ID3, as ID2’s genital area was not inspectable due to clothes. The examination did not enable the determination of these people’s sex, as the skin and muscle tissues of the pubic area were absent. Inspection of the facial skin did not reveal the presence of hair. Regarding age, the exposed bones did not show recent fusion areas between epiphyses and diaphyses, indicating that the subjects were at least of adult age, except for ID2, where the fusion line between the diaphysis and the proximal epiphysis of the tibia is still visible. This estimation was further supported and supplemented by the assessment of dental wear, suggesting for ID1 an adult-mature age (4th to 6th decades of life), for ID2 an adult age (3rd decade of life/1st half of the 4th), and for ID3 a mature-senile age (beyond the 6th decade of life). Bone trophism also aligns with age estimations. Concerning sex, morphological characteristics of the skulls suggest male sex for all three subjects, a hypothesis strongly supported by pelvic morphology and metric traits, some of which were estimable from AP and LL projections (43). Radiographic analysis revealed no signs of artificial embalming procedures in any of the examined mummies. We did not detect the presence of artificial eyes, eye caps, or tampons in the mouth, nasal passages, or rectum. None of the individuals examined showed any evidence of such practices (44).

Less radio-dense areas along the path of the middle meningeal artery, accompanied by a deep groove, characterize the parietal bones of ID1. The middle meningeal artery can form a groove on the inner skull surface. In some individuals, this groove may be more pronounced (45). This feature could suggest either a natural prominence of the artery or a thinning of the cranial bone in that region, both of which fall within normal anatomical variation and do not necessarily indicate pathology (46, 47). Other pathological possibilities, such as hyperostosis frontalis interna (excluded due to the absence of bone thickening) and infectious diseases like tuberculosis, should also be considered, though no additional radiographic signs support this (47–50). The same applies to the sequelae of meningitis, hematomas, neurovascular diseases, persistent arteritis, or hematomas (51–53). Given the subject’s sex and age, we cannot definitively rule out these other hypotheses.

Calcifications in the intervertebral discs between the 8th and 11th thoracic vertebrae, extending into the lumbar region, may indicate degenerative processes. Considering the subject’s age, these calcifications may be due to spondylosis, which affects about 80% of men by age 50 (54). The absence of osteophytes along the margins might suggest an early stage of the condition or a less aggressive form of spondylosis, where calcification is the primary or sole visible sign (55, 56). Infectious diseases such as tuberculosis or brucellosis, along with metabolic disorders like ochronosis, seem unlikely due to the lack of degeneration in both the vertebral endplates and bodies (57–60). Another possibility is that these calcifications result from age-related disc degeneration of idiopathic origin (61). Calcifications on the articular surfaces of the sternum and sacroiliac joints, alongside osteophytes in the pelvic joints, likely suggest a widespread degenerative joint condition (62, 63). The incomplete closure of the sacral laminae is probably related to a congenital condition known as spina bifida occulta, a relatively common malformation that is typically asymptomatic and does not usually cause problems during the individual’s lifetime (64). However, it is noteworthy that some studies have suggested a relationship between spinal anomalies associated with spondylosis and spina bifida occulta (65).

The denser bone striations observed in the distal diaphysis of both tibiae, approximately 1.7 cm from the distal articular surface, can be interpreted as Park-Harris lines, also known as growth arrest lines, which are frequently seen in individuals who experienced periods of physiological stress during growth, in this case around the ages of 6–7 years (66, 67).

Radiological evidence for ID1 presents a complex picture, suggesting an individual who lived with a combination of degenerative joint processes, congenital anatomical variations, and possible signs of physiological stress during growth. Given the subject’s sex and advanced age, it is likely that the degenerative evidence is attributable to both age and possibly repetitive weight-bearing activities, with the effects on the axial skeleton potentially exacerbated by genetic factors such as spina bifida occulta.

Regarding ID2, a dense network of venous impressions on the parietal bones and arachnoid granulations visible in the anteroposterior view may indicate a marked prominence of the superficial cerebral venous system (68, 69). This feature could be a sign of advanced age, during which progressive thinning of the cranial vault occurs, allowing for greater visibility of vascular structures (70). While this phenomenon can be observed in healthy individuals, it is important to rule out pathological conditions such as chronic intracranial hypertension and biparietal thinning, which could exacerbate these impressions (71, 72). However, in the absence of other signs of intracranial hypertension, such as erosion of the bone margins or enlargement of the sella turcica, this manifestation can be considered a result of the normal aging process (73). The expansion of the frontal sinuses toward the lateral aspect, nearly extending beyond the orbits, is an anatomical feature that can vary significantly among individuals (74). In elderly individuals, such pronounced expansion may be associated with progressive resorption of the frontal bone, which is consistent with the pneumatization process of the sinuses during aging (75, 76). No signs of chronic sinusitis or other inflammatory pathologies could explain this expansion, suggesting that this feature represents a normal anatomical variation rather than a pathological finding (77, 78).

Edentulism with alveolar tissue retraction is typical of advanced age. This condition is often associated with bone resorption processes, manifesting here as the retraction of the alveolar ridges (79). This process can accelerate in individuals who have been toothless for many years and may be associated with masticatory difficulties, nutritional challenges, and potential pre-existing oral infections (80).

The roughness observed in the left clavicle’s acromion, as in the insertion sites of the trapezius and subscapular muscles, suggests the presence of well-developed enthesopathies (81). These are often the result of repeated mechanical stress, exacerbated by obesity, which increases the load on joints and tendons (82). Calcifications in muscle insertion areas, such as the pectoralis major or capsular ligament, are consistent with calcific tendinopathy, a common degenerative disorder in old age that can cause chronic pain and limited range of motion (83, 84).

Intervertebral calcifications, fusion between the 8th and 9th thoracic vertebrae, and severe osteophytic changes with sclerosis of the vertebral endplates indicate advanced spondylosis (57). The vertebral fusion via a bony bridge is typical of ankylosis, which can be secondary to chronic degenerative or inflammatory processes such as ankylosing spondylitis (85). However, the absence of other typical features of ankylosing spondylitis, such as widespread enthesopathy and sacroiliac joint fusion, makes this diagnosis less likely (86–88). This degeneration is exacerbated by age and excess weight, contributing to the wear and tear of intervertebral joints and the development of osteophytes along vertebral margins (89, 90). Diffuse osteophytes in the pelvic girdle, particularly in the insertion areas of the oblique muscles on the iliac crest, the ischial tuberosity, and the iliopsoas muscle, as well as osteophytes along the margins of the patella and knee joints, suggests severe degenerative osteoarthritis of the lower appendicular skeleton (91, 92). This condition is common in overweight individuals, as excess weight exerts continuous pressure on weight-bearing joints, accelerating the degenerative process (93). The knee joints show signs of significant deformation, indicating that osteoarthritis had a substantial functional impact on this individual, likely limiting mobility and contributing to a progressive physical decline (94–96).

The spurs at the insertion of the Achilles’ tendon indicate insertional enthesopathy, a condition often associated with mechanical overload and repeated microtrauma (97). In an overweight individual, this condition could result from continuous stress on the tendon due to body weight and altered gait biomechanics (98). This condition may have caused pain and functional limitations, impacting the individual’s ability to walk and further contributing to a reduced quality of life (99).

ID2, an elderly overweight male over the age of 50, presents a profile of widespread degenerative pathologies reflecting the cumulative effects of advanced age, excess weight, and mechanical wear. The primary diagnosed conditions include advanced osteoporosis with diffuse osteophytosis, severe spondylosis, multiple enthesopathies, and significant osteoarthritis in weight-bearing joints. While most of the observed conditions can be attributed to aging and overweight, the severity and extent of the lesions suggest that this individual may have lived with chronic pain and significant mobility reduction in the later years of his life. The combination of these pathologies likely limited his ability to perform daily activities, contributing to a progressive decline in his quality of life.

The radiographic acquisitions of ID3 allow for the investigation of the pulp chamber morphology of the second lower molar. Micronutrient deficiency episodes during development are likely associated with this feature (100). Insufficient vitamin D, calcium, or phosphorus can influence dental and skeletal health, potentially manifesting as atypical tooth shapes and broader bone effects (101). The compression of the eighth thoracic vertebra could suggest a traumatic etiology; however, the degeneration of the disc endplates in this spinal region allows for a tentative hypothesis of an infectious disease like tuberculosis, which often presents with characteristic lesions in this anatomical location, although no other skeletal evidence supports this hypothesis (102–104). Additionally, the significant alterations at the insertion sites of the oblique muscles on the iliac crest suggest enthesopathy, which, given the subject’s age, is likely the result of mechanical stress.

The overall skeletal health condition of ID3 indicates what appears to be generally ‘good health’, with some episodes of nutritional deficiency during childhood. An area that warrants further investigation, given the subject’s age, is the mid-thoracic region, to gather additional information that could refine the differential diagnosis.

Given the data acquired from each discipline within this contribution, it is possible to highlight the abundant informational potential of conventional radiological techniques, an approach also emphasized in other studies. However, these techniques are still less frequently used compared to tomographic analyses (105–110).

## 5 Conclusion

This study highlights the potential of mobile digital radiographic technology in mummified or embalmed body examination. The radiographic acquisitions proved to be crucial across various disciplines, enabling the collection of data and the formulation of hypotheses that would otherwise be unattainable. The study emphasizes the importance of radiology for anthropological and paleopathological purposes and more specialized analyses, such as restoration history, taphonomy, clothing examination, and entomology. This approach underscores how radiographic analysis is an essential foundation in human remains analyses, serving as a non-destructive technique that should be among the first applied to such specimens. While acknowledging the significant role of CT scans, this study has demonstrated the value of X-ray radiography as a viable alternative, particularly considering the challenges and limitations associated with CT equipment on mummified or embalmed remains.

## Data Availability

The raw data supporting the conclusions of this article will be made available by the authors without undue reservation.
