# Correction: Integrated multidisciplinary analysis of mobile digital radiographic acquisitions of the mummies of the Hermits from the Sanctuary of Madonna della Corona (Italy – 17th to 19th Century CE)

**DOI:** 10.3389/fmed.2025.1736955

**Published:** 2025-11-17

**Authors:** Omar Larentis, Ilaria Gorini, Michele Campus, Marta Lorenzetti, Giancarlo Mansueto, Susanna Bortolotto, Emanuele Zappa, Andrea Gregorini, Laura Rampazzi, Stefano Vanin, Giuseppina Carta, Alberto Carli, Lara Simonaitis, Lisa De Luca, Enrica Tonina

**Affiliations:** 1 CROP - Centre of Research in Osteoarchaeology and Paleopathology, Biotechnologies and Life Sciences Department (DBSV), University of Insubria, Varese, Italy; 2 LaBAAF - Bagolini Laboratory of Archaeology, Archaeometry, Photography, Department of Humanities, University of Trento, Trento, Italy; 3 Superintendence for Archaeology, Fine Arts, and Landscape for the provinces of Como, Lecco, Monza-Brianza, Pavia, Sondrio and Varese, Milan, Italy; 4 Fujifilm Italia, Cernusco sul Naviglio, Italy; 5 Studio di conservazione tessile Lorenzetti, Verona, Italy; 6 Diagnostics and Public Health Department (DDSP), University of Verona, Verona, Italy; 7 Department of Architecture and Urban Studies, Polytechnic University of Milan, Milan, Italy; 8 Department of Mechanical Engineering, Polytechnic University of Milan, Milan, Italy; 9 Department of Human Sciences and Innovation for the Territory, University of Insubria, Como, Italy; 10 Earth, Environment and Life Sciences Department (DISTAV), University of Genoa, Genoa, Italy; 11 SUSeF, Department of Humanities, Social Sciences and Education, University of Molise, Campobasso, Italy

**Keywords:** X-ray analyses, anthropology, paleopathology, entomology, restoration, conservation, taphonomy, ethics

The title of this article was erroneously given as “Integrated multidisciplinary analysis of mobile digital radiographic acquisitions of the mummies of the Hermits from the Sanctuary of Madonna della Corona (Trentino-Alto Adige, Italy - 17th to 19th Century CE)” The correct title of the article is “Integrated multidisciplinary analysis of mobile digital radiographic acquisitions of the mummies of the Hermits from the Sanctuary of Madonna della Corona (Italy - 17th to 19th Century CE)”.

The original version of this article has been updated.

